# A computed tomography based survey study investigating the agreement of the therapeutic strategy for fragility fractures of the pelvis

**DOI:** 10.1038/s41598-022-04949-x

**Published:** 2022-02-11

**Authors:** Philipp Pieroh, Tim Hohmann, Florian Gras, Sven Märdian, Alexander Pflug, Silvan Wittenberg, Christoph Ihle, Notker Blankenburg, Kevin Dallacker-Losensky, Tanja Schröder, Steven C. Herath, Hans-Georg Palm, Christoph Josten, Fabian M. Stuby, Daniel Wagner, Andreas Höch

**Affiliations:** 1 Department of Orthopedics, Trauma and Plastic Surgery, University of Leipzig, Liebigstrasse 20, 04103 Leipzig, Germany; 2 Department of Anatomy and Cell Biology, Martin Luther University Halle-Wittenberg, Halle (Saale), Germany; 3 Department of Trauma-, Hand- and Reconstructive Surgery, University Hospital Jena, Jena, Germany; 4 Centre for Musculoskeletal Surgery, Charité-University Medicine Berlin, Berlin, Germany; 5 BG Trauma Center, Eberhard Karls University, Tuebingen, Germany; 6 Trauma Research Group, Department of Orthopedics and Trauma Surgery, Reconstructive and Septic Surgery, and Sports Traumatology, Bundeswehrhospital Ulm, Ulm, Germany; 7 Department of Trauma, Hand and Reconstructive Surgery, Saarland University Hospital, Homburg, Germany; 8 Department of Trauma Surgery, BG Trauma Centre Murnau, Murnau am Staffelsee, Germany; 9 Department of Orthopedics and Traumatology, University Medical Center Mainz, Mainz, Germany; 10 Present Address: Address: Department of Orthopedic and Trauma Surgery, University Hospital Erlangen, Erlangen, Germany; 11 German Pelvic Injury Register, German Society of Traumatology, Berlin, Germany

**Keywords:** Geriatrics, Fracture repair, Reconstruction

## Abstract

Treatment recommendations for fragility fractures of the pelvis (FFP) have been provided along with the good reliable FFP classification but they are not proven in large studies and recent reports challenge these recommendations. Thus, we aimed to determine the usefulness of the FFP classification determining the treatment strategy and favored procedures in six level 1 trauma centers. Sixty cases of FFP were evaluated by six experienced pelvic surgeons, six inexperienced surgeons in training, and one surgeon trained by the originator of the FFP classification during three repeating sessions using computed tomography scans with multiplanar reconstruction. The intra-rater reliability and inter-rater reliability for therapeutic decisions (non-operative treatment vs. operative treatment) were moderate, with Fleiss kappa coefficients of 0.54 (95% confidence interval [CI] 0.44–0.62) and 0.42 (95% CI 0.34–0.49). We found a therapeutic disagreement predominantly for FFP II related to a preferred operative therapy for FFP II. Operative treated cases were generally treated with an anterior–posterior fixation. Despite the consensus on an anterior–posterior fixation, the chosen procedures are highly variable and most plausible based on the surgeon’s preference.

## Introduction

**Figure 1:**
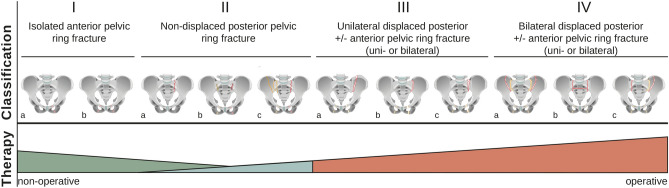
Fragility fracture of the pelvis (FFP) classification. The FFP classification is outlined according to the characteristic fracture morphology. The main lesions are in red and the less common lesions are in orange. Non-operative treatment is recommended for FFP I and FFP II. Operative stabilization is recommended for FFP III and FFP IV. FFP II with prolonged pain or restricted mobilization should be considered for operative treatment as well.

Fragility fractures of the pelvis have increased in recent years, accompanied by the loss of mobility and autonomy, with increased rates of mortality^1–5^. Variable morphology, dynamic fracture progression, and the resulting instability have led to the development of a computed tomography (CT)-based FFP classification^6^ (Fig. 1) which is as reliable as the OTA/Tile and Young and Burgess classifications^7,8^.

Based on the classification the following recommendations were given, non-operative treatment for FFP I; non-operative or operative treatment, depending on the patient’s mobility, for FFP II; and operative treatment for FFP III and FFP IV^9^.

However, recent monocentric studies challenged the recommendations of Rommens and Hofmann^6^ and showed good results for operatively treated FFP II^10–13^ and non-operatively treated FFP III and FFP IV^14,15^.

This brings into question the usefulness of the FFP classification and the accompanying treatment recommendations.

Currently, there is a lack of data on the usefulness of the FFP classification for therapeutic decision-making, even though this data is essential for preventing harm that could occur due to inappropriate classification of the fracture categories that may lead to incorrect treatment decisions^16^.

Therefore, in the present study we used CT with multiplanar reconstruction, without clinical information and assessed the intra-rater reliability and inter-rater reliability of therapeutic decision-making for FFP (non-operative vs. operative treatment). We investigated the treatment recommendations, their relation to the FFP classification^6^ and the effects of classification disagreement on treatment decisions.

Using this approach, we aimed to determine the reliability of treatment strategies derived from CT scans and resulting FFP classification, and thus the usefulness of FFP classification in relation to clinically relevant decisions (operative vs. non-operative). Furthermore, we investigated the favored operative procedures in relation to the FFP classification.

## Methods

### Study design

The study design used to evaluate the FFP classification, sample size calculation, patient demographics, anonymization, and rating procedures was reported by Pieroh et al.^8^ The patients from this study^8^ were used to analyze the association between the FFP classification and the resulting treatment decision as well as the favored operative procedure. Each observer was familiar with the FFP classification and no additional training was performed before the study. At least two weeks lay between the classification cycles and observers had no access to the previous ratings and classification cycles.

**Figure 2:**
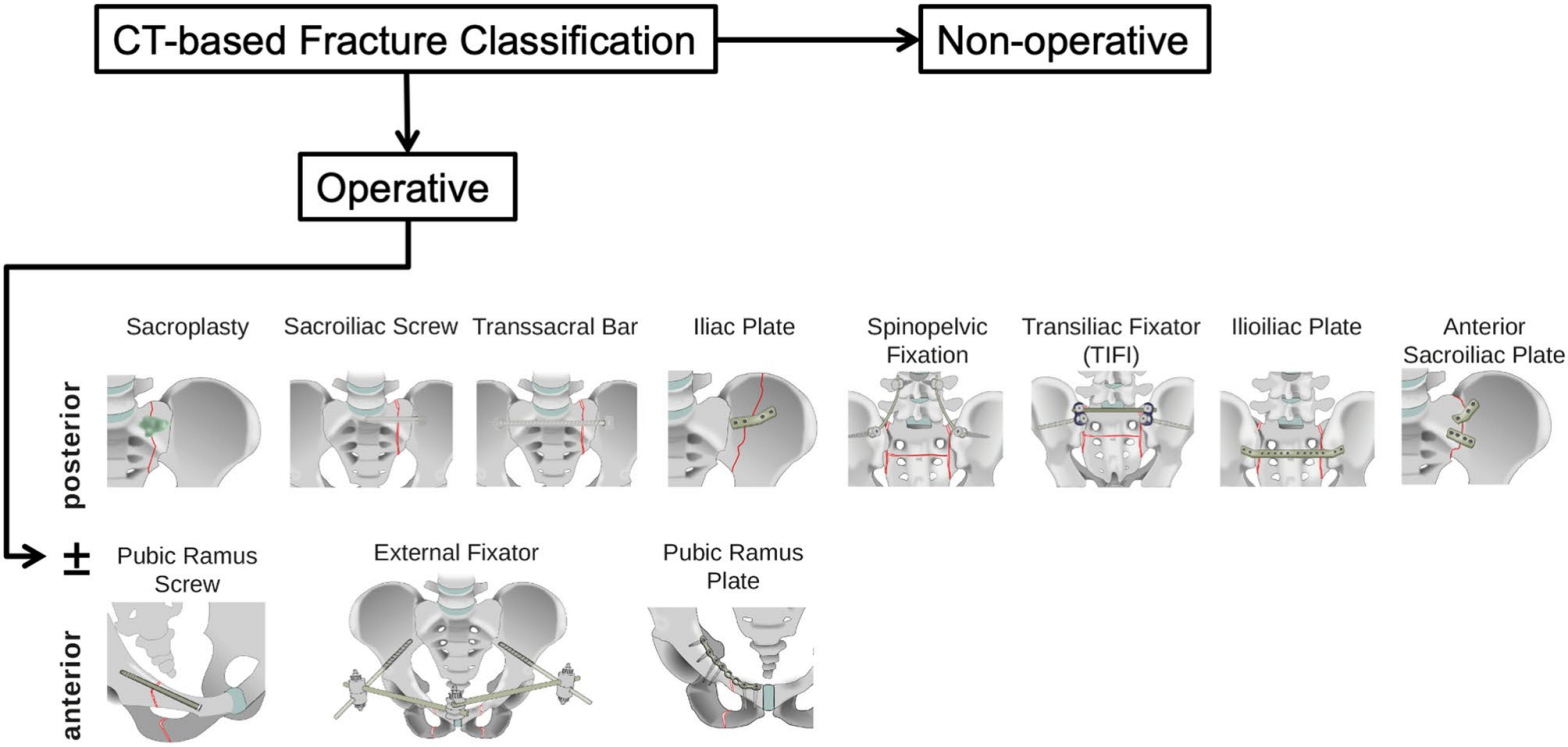
Treatment decisions for FFP. At first, the rater had to decide between non-operative and operative treatment. No further specific treatment data were obtained when non-operative treatment was performed. For operative treatment, the rater had to decide whether to use anterior and/or posterior stabilization. For anterior stabilization, the rater could choose between procedures; no combinations were possible. For posterior stabilization, the rater could choose unilateral or bilateral stabilization and further specified the operative method; combinations were possible.

In addition to classification, recommended treatment options are also presented in Fig. 2.

### Statistical analyses

#### Intra-rater reliability and Inter-rater reliability

We assessed the intra-rater reliability and inter-rater reliability of the therapeutic decisions (non-operative vs. operative) as previously reported^8^ using specific MATLAB scripts (MATLAB, version 2013b; MathWorks, Natick, MA, USA) to calculate the Fleiss kappa coefficients^17,18^ and presented them as means and 95% confidence intervals (CI). The 95% CI was generated using the bootstrap method of resampling the pelves^17,18^.

Treatment decisions were collected during the classification process from each of the 6 experienced and inexperienced surgeons and the surgeon trained by the creator of the FFP classification (“gold standard”), all from Level-1 trauma centers^8^. Inexperienced raters were included as part of this pragmatic multicenter agreement study to assess the generalizability of the classification related decisions in raters with differing experience^16^.

The “gold standard” was included due to his adherence to the prescribed treatment recommendations^6^. We generated the intra-rater reliability based on the three classification cycles, separate for each rater. For the inter-rater reliability, we calculated one mean vote for each rater out of the three classification cycles and used this for further analysis. Using this data, we determined the inter-rater reliability for each classification cycle and for the overall cycles.

We graded the intra-rater reliability and inter-rater reliability using the following categories determined by Landis and Koch^19^: Fleiss kappa coefficient of 1, perfect reliability; ≥ 0.81, almost perfect reliability; 0.61–0.80, substantial reliability; 0.41–0.60, moderate reliability; 0.2–0.40, fair reliability; and ≤ 0.21, poor reliability.

#### Agreement analyses

We used the classifications and therapeutic decisions determined by the references, “gold standard”, submitting hospitals, and majority vote. We investigated the agreement between the therapeutic decisions of the raters and the therapeutic decisions indicated by the references for FFP. We generated the majority vote, similar to the mean vote, for calculating the inter-rater reliability and included the gold standard and submitting hospital votes.

#### Classification and therapeutic decision agreement

Case 48 was excluded because its FFP classification was not possible according to the gold standard and 42.9% of raters^8^. We generated the majority vote based on the rater votes for the classification and therapeutic decision for each case (n = 59). The gold standard and submitting hospital votes were excluded from this vote. Using the therapeutic decision of that majority vote, we separated cases according to the recommended non-operative and operative treatments. Subsequently, we allocated cases to the FFP classification based on the gold standard classification. We examined the agreement and disagreement (“gold standard” vs. raters) between the classification and therapeutic decision for each case to assess the impact of classification disagreement on the therapeutic decision.

#### Preferred operative therapy

Cases where operative therapy was recommended, were analyzed to assess the operative therapy (Fig. 2) preferred by all the raters, the gold standard, and the submitting hospital. Raters had to choose an anterior procedure. For posterior stabilization, unilateral or bilateral procedures could be chosen. The available procedures could be combined.

### Ethical statement and study registration

The following ethics committees approved the study: Ethics Commission at the Medical Faculty of the University of Leipzig, Universitäts Klinikum Jena Ethics Commission, Ethics Committee Charité-Universitätsmedizin Berlin, Ethics Commission Medical Association of Saarland, Ethics Committee at the Medical Faculty of the Eberhard Karls University and at the University Hospital Tübingen, Ethics Committee of the University of Ulm.

All these mentioned ethics committees waived the need for informed consent of the patients for this study due to the retrospective nature of the study and because patients consented to the use of de-identified CT scans for research on signing the hospital contract of admission. Separate informed consent was not obtained since the data was collected retrospectively. Afterwards, the study was registered in the German Clinical Trials Register (DRKS00014248). CT scans with multiplanar reconstruction were performed for clinical reasons. The study was performed in accordance to the Declaration of Helsinki.

## Results

Classifications and treatment decisions based on the gold standard, submitting hospital, and majority vote for each patient, are summarized in Supplementary Table S1. The patient demographics are available in Appendix II of Pieroh et al.^8^.

### Intra- and Inter-rater reliability

The gold standard and both raters of hospital 1 had almost perfect intra-rater reliability (Table 1). The decisions of two experienced and three inexperienced raters had substantial intra-rater reliability. The overall intra-rater reliability (Table 1), overall inter-rater reliability and inter-rater reliability of the experienced raters were moderate (Table 2). The overall inter- rater reliability of the decisions of the inexperienced raters was fair (Table 2).

**Table 1:** Intra-rater reliability of the therapeutic decision (non-operative vs. operative) for the three classification cycles.

	Mean Fleiss Kappa coefficient (95% CI)		
	Experienced	Inexperienced	“Gold standard”
	Mean [95% CI]	Mean [95% CI]	Mean [95% CI]
**Hospital**			
1	0.86 [0.75;0.96]	0.95 [0.88;1]	
2	0.73 [0.58;0.85]	0.68 [0.53;0.82]	
3	0.49 [0.31;0.63]	0.60 [0.43;0.75]	
4	0.51 [0.32;0.67]	0.70 [0.55;0.84]	
5	0.56 [0.39;0.73]	0.52 [0.32;0.70]	
6	0.76 [0.63;0.89]	0.72 [0.58;0.86]	
Overall	0.58 [0.48;0.67]	0.51 [0.40;0.60]	0.85 [0.73;0.95]

**Table 2:** Inter-rater reliability of the therapeutic decision (non-operative vs. operative) for all classification cycles and for each separate cycle.

	Mean Fleiss Kappa coefficient (95% CI)		
	Overall	Experienced	Inexperienced
**Cycle**			
1st	0.54 [0.43;0.64]	0.59 [0.47;0.70]	0.47 [0.34;0.58]
2nd	0.55 [0.44;0.64]	0.59 [0.48;0.69]	0.48 [0.35;0.60]
3rd	0.52 [0.41;0.62]	0.53 [0.42; 0.65]	0.46 [0.34;0.58]
Overall	0.42 [0.34;0.49]	0.51 [0.42;0.58]	0.31 [0.23;0.37]

### Agreement analyses

The highest therapeutic agreement (> 90%) was found for FFP I and the lowest was found for FFP II (minimum compared to the gold standard, 66.0%) (Table 3). For FFP I and FFP II, the majority voted for non-operative therapy (Table 3). The agreement for FFP III and FFP IV was > 75%, and the majority recommended operative treatment.

**Table 3:** Percentage of agreement between raters and references (“Gold Standard,” submitting hospital, and majority vote).

	Mean % agreement (95% CI)									
	FFP main group	n	Experienced				Unexperienced			
			Non-operative	n	Operative	n	Non-operative	n	Operative	n
"Gold Standard" n = 59	I	11	98.5 [95.5;1]	11	–	0	95.5 [90.9;1]	11	–	0
	II	26	66.0 [53.2;79.5]	26	–	0	68.6 [58.3;78.9]	26	–	0
	III	9	–	0	96.3 [90.7;1]	9	–	0	92.6 [85.2;1]	9
	IV	13	–	0	83.3 [69.2;94.9]	13	–	0	84.6 [69.2;96.2]	13
Submitting hospital n = 60	I	13	93.6 [84.6;98.7]	13	–	0	91.0 [84.6;96.2]	13	–	0
	II	17	78.2 [71.8;84.6]	15	45.8 [0;91.7]	2	75.6 [61.5;87.2]	15	41.7 [8.3;79.2]	2
	III	13	8.3 [0;25.0]	3	83.3 [64.8;96.3]	10	20.8 [0;41.7]	3	81.5 [64.8;94.4]	10
	IV	17	43.3 [10.0;73.3]	5	76.4 [52.8;95.8]	12	43.3 [10.0;76.7]	5	76.4 [55.6;91.7]	12
Majority vote n = 59	I	12	98.6 [95.8;1]	12	–	0	94.4 [90.3;98.6]	12	–	0
	II	21	84.3 [77.5;90.2]	20	66.7 [41.7;91.7]	1	82.4 [75.5;89.2]	20	54.2 [37.5;75.0]	1
	III	10	–	0	96.7 [91.7;1]	10	–	0	91.7 [85.0;98.3]	10
	IV	16	55.6 [50.0;66.7]	3	92.3 [83.3;98.7]	13	66.7 [50.0;83.3]	3	92.3 [85.9;97.4]	13

### Classification and therapeutic decision agreement

**Figure 3:**
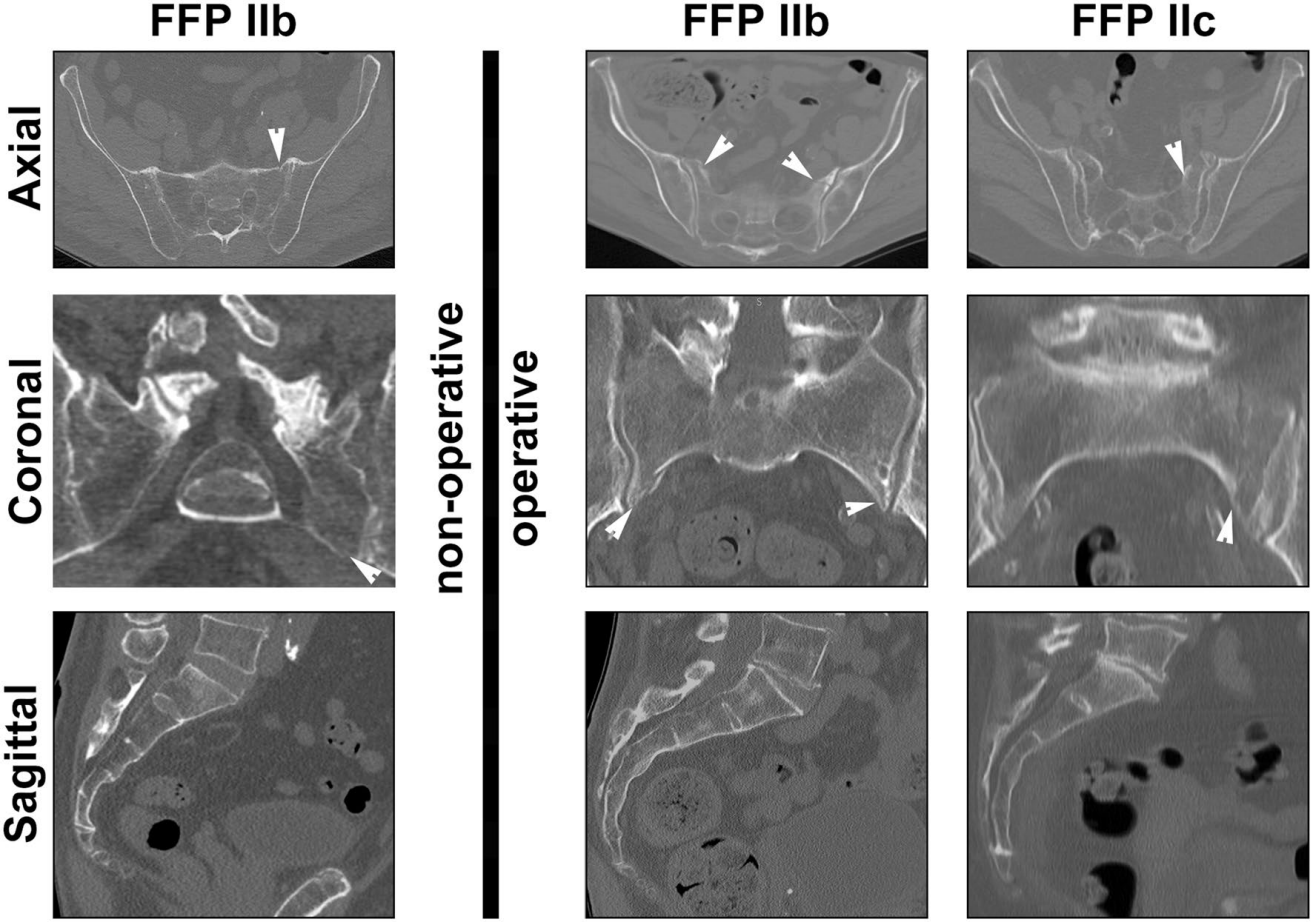
FFP II cases with classification agreement but differences in treatment recommendations. One FFP IIb case (non-displaced fracture of the sacral ala; anterior fracture not shown) was recommended to undergo non-operative treatment by the gold standard, submitting hospital, and raters. A bilateral non-displaced fracture of the sacral ala without horizontal communication (FFP IIb) was recommended to undergo operative treatment by the raters only. A unilateral, multi-fragmentary, non-displaced fracture of the sacral ala (FFP IIc) was recommended to undergo surgery by the raters and the submitting hospital. Fracture lines are indicated by white arrows.

For FFP I, FFP III, and FFP IV, the classifications and resulting treatment decisions were majorly in agreement (Table 4). Pronounced disagreement regarding therapy was found for FFP II, both in classification and treatment recommendation. Although the raters and gold standard agreed on the classification of one FFP IIb case and four FFP IIc cases, the raters recommended surgery (Fig. 3).

**Table 4:** Case-based (dis)agreement analysis of therapy (separation based on the majority vote) and classification (separation based on mean vote of the "Gold Standard").

FFP classification		Classification agreement [n]		Treatment agreement [n]	
		Agreement	Disagreement	Agreement	Disagreement
Non-Operative (n = 29)	Ia (n = 10)	10	–	10	–
	Ib (n = 1)	1	–	1	–
	IIa (n = 3)	3	–	3	–
	IIb (n = 12)	10	2	12	–
	IIc (n = 2)	–	2	2	–
	IVb (n = 1)	1	–	–	1
Operative (n = 30)	IIa (n = 2)	–	2	–	2
	IIb (n = 1)	1	–	–	1
	IIc (n = 6)	4	2	–	6
	IIIa (n = 6)	6	–	6	–
	IIIb (n = 1)	1	–	1	–
	IIIc (n = 2)	2	–	2	–
	IVb (n = 10)	10	–	10	–
	IVc (n = 2)	2	–	2	–

### Surgical treatment preferences

One FFP IIc case (case 39) was excluded from further analyses because < 50% recommended anterior stabilization and/or posterior stabilization. The FFP classification, agreement regarding anterior stabilization and unilateral or bilateral stabilization, and the procedure frequencies are summarized in Supplementary Table S2. Anterior stabilization was recommended for 18 cases (Table 5, Supplementary Table S2). The external fixator was the favored anterior procedure. Posterior instrumentation was recommended for all cases with surgical stabilization. Bilateral fixation was recommended for FFP IIa, FFP IIb, FFP IIIb, and FFP IV cases, which corresponded to 58% (n = 17) of all cases recommended for surgery (Table 5).

**Table 5:** Preferred surgical therapy (anterior and posterior) in relation to the FFP classification.

	Frequencies [n]					
	Anterior stabilization		Posterior stabilization			
			Uni-/bilateral [n]?		Favored Procedure and number of cases [n]	2nd choice and number of cases [n]
			Unilateral	Bilteral		
	Amount [n]	Favored Procedure and number of cases [n]				
IIa (n = 2)	–	–	–	2	SIS/TSB n = 2	SPF, n = 2
IIb (n = 1)	–	–	–	1	SIS/TSB n = 1	TIFI, n = 1
IIc (n = 5)	3	EF, n = 3	4	1	SIS/TSB n = 4; IP n = 1	TIFI, n = 2; SIS + IP, n = 2; SPF, n = 1
IIIa (n = 6)	5	EF, n = 3; EF or PO, n = 1; EF or SO, n = 1	6	–	IP, n = 6	ASP, n = 1; SPF, n = 1; SIS/TSB + IP, n = 1; SIS/TSB, n = 1; IP + ASP, n = 2;
IIIb (n = 1)	1	PO, n = 1	–	1	SIS/TSB + IP, n = 1	SPF, n = 1
IIIc (n = 2)	2	EF, n = 1; PO, n = 1	2	–	SIS/TSB, n = 2	TIFI, n = 2
IVb (n = 10)	5	EF, n = 4; PO n = 1	–	10	SIS/TSB, n = 5; SPF, n = 5	SIS/TSB + IP, n = 4; SPF, n = 5; SIS/TSB + SPF, n = 1
IVc (n = 2)	2	EF, n = 1; PO, n = 1	–	2	SIS/TSB + IP, n = 1; SPF + IP, n = 1	SIS/TSB + IP, n = 1; SPF + IP, n = 1

*EF* external fixator, *PO* plate osteosynthesis, *SO* screw osteosynthesis, *SIS* sacroiliac screw, *TSB* transsacral bar, *SPF* spinopelvic fixation, *TIFI* trans-iliac fixator, *IP* iliac plate through lateral window of the ilioinguinal approach, *ASP* anterior sacoriliac plate.

**Figure 4:**
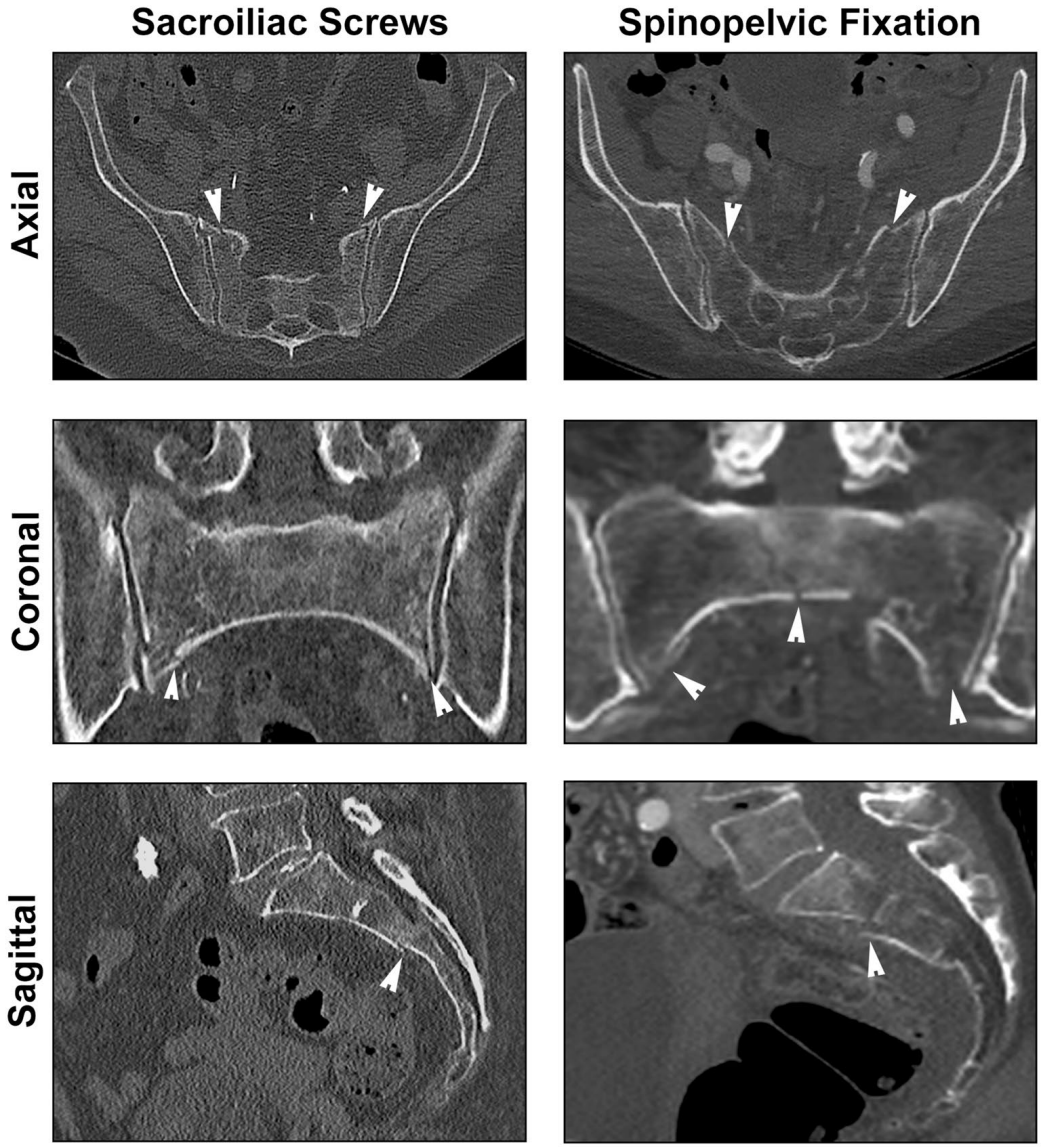
FFP IVb examples with differing recommended posterior operative stabilization methods. Approximately half of the raters recommended that the presented fractures required sacroiliac screws (SIS) or spinopelvic fixation (SPF) (maximum rating difference, 2 votes). The fracture recommended for SIS was a bilateral non-displaced fracture of the sacral ala with vertical communication below S2 and minimal anterior displacement. The fractures recommended for SPF were a displaced trans-foraminal fracture (Denis zone II), a non-displaced fracture of the sacral ala, and a central fracture through S1. In the sagittal view, the vertical fracture through S1 without anterior or posterior displacement is revealed.

Unilateral stabilization was predominantly recommended for FFP IIc, FFP IIIa, and FFP IIIc. For sacral fractures (FFP II and FFP IIIc), the raters preferred stabilization with sacroiliac screws/transsacral bar or, as second choice, with a trans-iliac fixator or spinopelvic fixation. Transiliac fractures (FFP IIIa) were treated with an iliac plate through the lateral window using the ilioinguinal approach. Sacroiliac screws/transsacral bar and an iliac plate were recommended for the FFP IIIb case. Half of the FFP IVb cases were recommended to undergo treatment with sacroiliac screws/transsacral bar, and the other half of the FFP IVb cases were recommended to undergo spinopelvic fixation (Fig. 4). For FFP IVb, if sacroiliac screws/transsacral bar fixation was chosen, then the second choice was spinopelvic fixation, and vice versa.

Anterior and posterior, percutaneous procedures were the preferred choice for operatively treating FFP.

## Discussion

The surgeons agreed to treat isolated anterior lesions (FFP I) non-operatively and to recommend operative treatment for posteriorly displaced fractures (FFP III and FFP IV). There was some disagreement regarding the therapeutic decisions for non-displaced posterior fractures (FFP II). For cases with indications for operative treatment, a combination of anterior and posterior surgery was recommended.

Classification systems should distinguish patients receiving non-operative or operative treatment. Despite a high reported agreement in treating LC-1 fractures/Tile B fractures^20^ (comparable to FFP II)—representing the most common fracture type in the elderly^21^—the disagreement found in the survey analysis of Beckmann et al.^22^ highlights the differing treatment for a similar fracture type. To improve the treatment, the examination under anesthesia (EUA) for lateral compression fractures was introduced, but their interpretation shows a relevant disagreement^23^. Furthermore, the deduced treatment and the consensus might change upon newer data^24–26^.

The moderate intra-rater reliability and inter-rater reliability for therapeutic decisions observed during our study might be the result of still conflicting information about mortality after non-operative or operative treatment for FFP, and some evidence of lower mortality for operated cases^8,27,28^. Here, we would like to emphasize that the grading was done using the arbitrarily, not proven but widely used benchmarks according to Landis and Koch^29^. Using the stricter and probably more practical^29^ but also arbitrarily set ranges of Svanholm et al.^30^ would lead to good intra-rater reliabilities for all raters but only good inter-rater reliabilities for experienced observers. The missing validation of these criteria should be considered weighting the presented Fleiss kappa values.

The inexperienced raters presented only a fair inter-rater reliability. One reason might their missing experience in treating pelvic ring injuries by their own. However, in our view most probably, the differences result from classification differences especially between non-displaced and displaced posterior pelvic ring lesions (FFP II vs. FFP III) and the missing detection of the horizontal connection of bilateral sacral lesion leading to a difference in treatment from non-operative to operative treatment.

Therapeutic decisions for FFP based on morphology or classification have not yet been studied, although fracture severity (Tile A vs. B) influenced the survival after FFP^31^. Similar to a survey analysis on the treatment of high-energy pelvic fractures, we determined a high agreement for stable (Tile A—FFP I) and completely unstable fractures (Tile C—FFP III, FFP IV) but a low agreement for partially stable injuries (Tile B—FFP II)^20^.

Our study showed a consensus for the treatment of FFP I, FFP III, and FFP IV by applying the consensus criteria (agreement ≥ 75%) for Delphi studies^32^. The therapeutic strategy chosen by the raters generally followed the recommendations of Rommens and Hofmann^6,33^. Fractures classified as FFP II showed the lowest agreement for the treatment strategy, probably because of the impaired differentiation between FFP II and FFP III^8^. However, the classification disagreement itself was not responsible for the differences in therapeutic decisions for FFP. The observers tended to recommend operative treatment for FFP II more often, probably due to the successful operative treatment of these injuries in terms of preserved autonomy, lower mortality^3^, and to hasten pain relief^10–12,34^. Furthermore, even incomplete, simple sacral fractures (FFP II) displace in approximately 30% of elderly patients to FFP III or FFP IV, especially in patients treated non-operatively, leading to a secondary operative procedure^35^. Even after 6 months of failed non-operative therapy, patients with FFP II benefited from operative therapy^10^. However, late surgical therapies might be complicated by deformities in contrast to early performed percutaneous procedures^6,36^.

Thus, a close follow-up especially for non-operatively treated cases is necessary. Additional factors such as general health^21^ and a radiographic rating system^37^ may help with decision-making, especially for FFP II.

Although our study indicated equal use of spinopelvic fixation^6^ and sacroiliac screws/transsacral implant fixation^38,39^ for posterior bilateral displaced sacral fractures (FFP IVb), discriminating radiological factors were not found. Significant pain reduction without complications after operative treatment using percutaneous transiliac-transsacral screws or bilateral sacroiliac screws in the upper sacral segment has been reported^38,40,41^. Interestingly, even unilateral injuries were recommended bilateral stabilization, probably to avoid fracture progression^35,42^.

The external fixator was preferred for the anterior ring, by four of the seven hospitals. The external pelvic fixator has been reported as a valuable tool for the elderly^43^, but it may be complicated by frequent loosening or pin tract infections^44,45^. Retrograde transpubic screw fixation has yielded good results in terms of fracture reduction and healing, with only a small number of adverse events (17 of 128 cases) reported by a large retrospective series^46^. Anterior plate osteosynthesis is complicated by loosening and consecutive non-union as well as excessive blood loss in elderly patients^47,48^. It should be noted that two hospitals and the gold standard preferred retrograde pubic screws whereas four hospitals preferred the external fixator.

Currently, it remains unclear which patients require anterior stabilization. A recent systematic review highlights the low number of cases treated anteriorly but also emphasizes the low quality of studies^49^. However, comparing all three anterior stabilization procedures, all of them showed a relevant complication rate, a maximum of one quarter of patients required revision surgery^46,47,50^. The need for anterior stabilization as well as the type of osteosynthesis should be investigated in prospective studies.

Clinical data regarding mobility, pain, perioperative risks, and expectations, especially for patients with FFP II is needed to make appropriate treatment decisions. The influences of these factors on the patient’s prognosis need to be elaborated^21^.

Besides, an incorrect classification of FFP II by the observer, the observer might decide to not follow the recommendations of Rommens and Hofmann^9^. This might be the result of current studies showing a decreased rate of mortality^51^, rate of general complications for percutaneous procedures^52^ and an improved mobility following operative therapy^53^. This data underlines the need to introduce additional modifiers for treatment decision (e.g. previous mobility level, pain level) and probably of a progressive operative treatment to avoid immobility-associated complications^54^. On the other hand, data from Saito et al. challenge the more progressive operative treatment^55^. Based upon the ongoing data gain, an adaption of the recommendations should be considered.

Non-operative therapy should be standardized and the time to failure of non-operative therapy should be defined to avoid immobility-associated complications such as pressure ulcera^54^.

The FFP classification has sufficient intra-rater and inter-rater reliability however the choice of fixation among the options available especially in the posterior ring injuries are somewhat unclear and depend more on the physician's experience and training.

Although we determined a high agreement of the raters to the proposed therapy by the FFP classification, the clinical course and success in patients must be proven.

The FFP classification might become useful for guiding non-operative and operative therapy strategies, but the inclusion of non-radiological data and recent studies is required to improve therapy guidance. Recommendations for treating FFP have not yet been finalized and controversy exists, especially concerning FFP II. Here, additional factors e.g. secondary displacement and/or unrelenting pain on ambulation should be considered in the change from non-operative treatment to operative treatment in patients with FFP II.

Most differences in procedure were observed due to the individual preference of a surgeon and missing evidence in terms of comparative studies. Based on these findings, the relevance of the FFP classification criteria, operative procedures, and their outcomes should be evaluated further. Here, the patients suffering from a FFP II requiring operative treatment, the concept for non-operative therapy, the most non-invasive type of stabilization with the required stability, fractures at risk for fracture progression as well as the patients requiring anterior fixation should be identified.

## Supplementary Information

Supplementary Information.

## Data Availability

The datasets generated and analyzed during the current study are available from the corresponding author on reasonable request.
