# Supplementary material for: A computed tomography based survey study investigating the agreement of the therapeutic strategy for fragility fractures of the pelvis
Source: Sci Rep. 2022 Feb 11;12:2326. doi: 10.1038/s41598-022-04949-x (PMC8837654; doi:10.1038/s41598-022-04949-x)

**Therapeutic Strategy for Fragility Fractures of the Pelvis: Intra-rater and Inter-rater Reliability, Agreement, and Classification Analyses**

Philipp Pieroh^1,2+*^, Tim Hohmann^2^, Florian Gras^3+§^, Sven Märdian^4+^, Alexander Pflug^3^, Silvan Wittenberg^4^, Christoph Ihle^5^, Notker Blankenburg^1^, Kevin Dallacker-Losensky^6^, Tanja Schröder^7^, Steven C. Herath^5,7+^, Hans-Georg Palm^6+¥^, Christoph Josten^1+^, Fabian M. Stuby^8+#^, Daniel Wagner^9­­¶+^, Andreas Höch^1¶+^

Supplementary Table S1: Classification and Therapeutic decision for each case by the "Gold Standard", submitting hospital and majority vote. For the majority vote, the percentage of agreement is given in brackets.


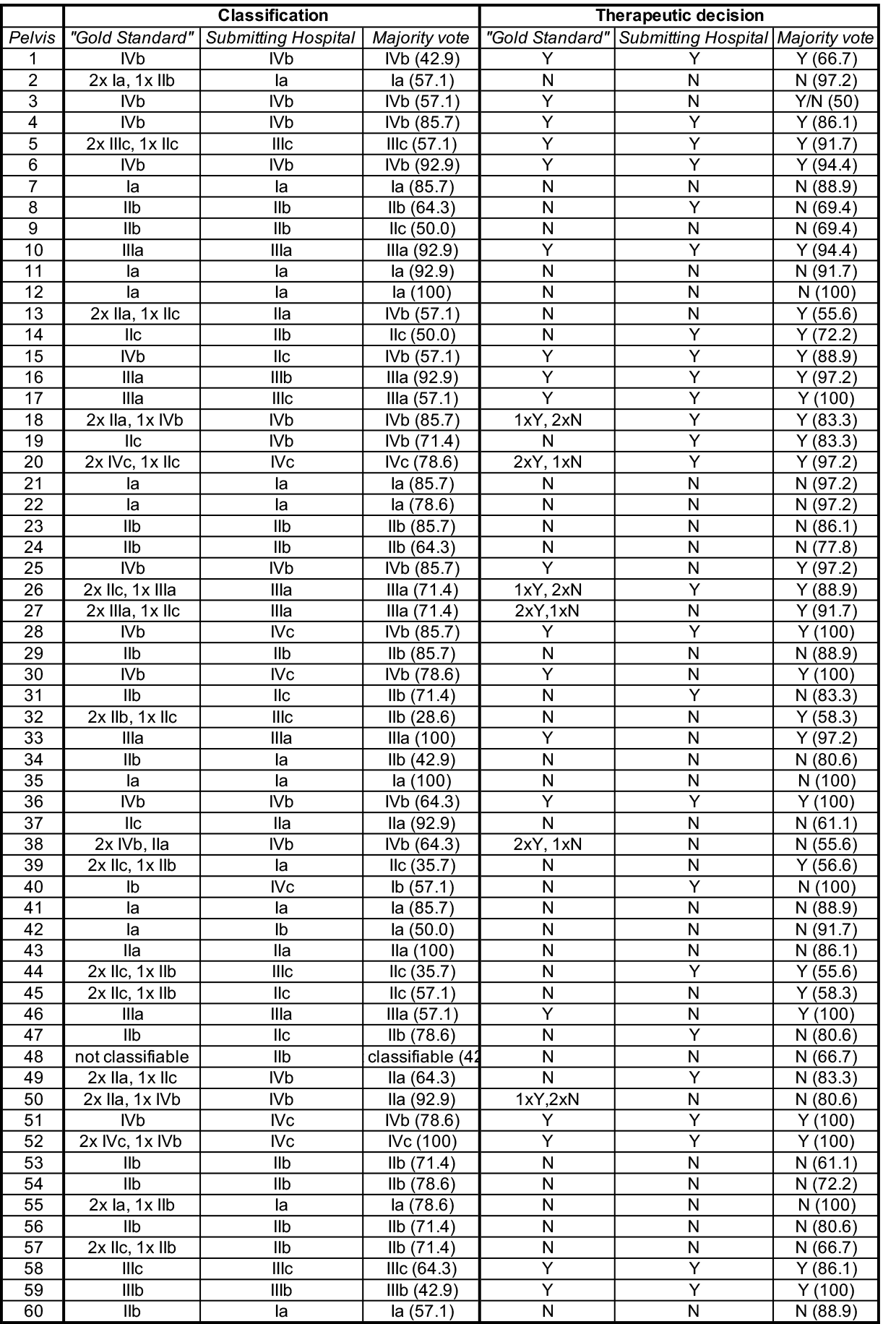


Supplementary Table S2: FFP Classification, percentage of agreement for anterior stabilization with favored procedure and votes for the techniques, decision between uni- or bi-lateral treatment with the percentage of agreement, posterior favored technique with total votes and the percentage of votes, remaining procedures with amount of votes in decreasing frequency.


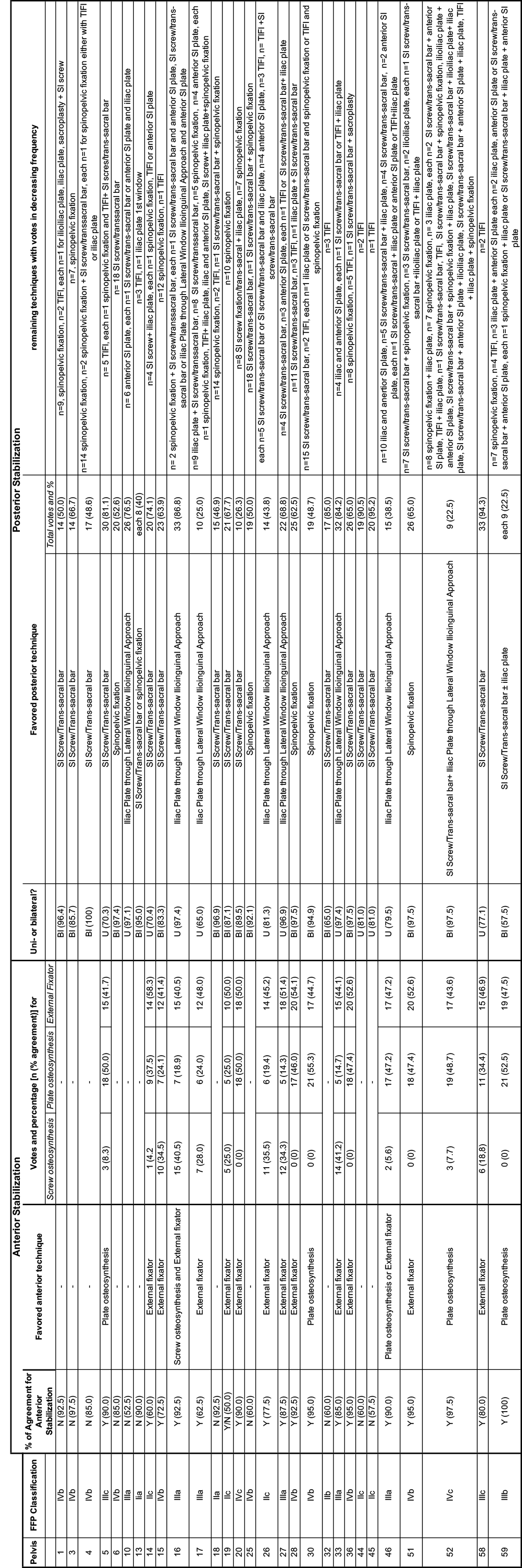

Supplement: Supplementary file 1 — Supplementary Information. [file 41598_2022_4949_MOESM1_ESM.docx]
